# Using dropout based active learning and surrogate models in the inverse viscoelastic parameter identification of human brain tissue

**DOI:** 10.3389/fphys.2024.1321298

**Published:** 2024-01-23

**Authors:** Jan Hinrichsen, Carl Ferlay, Nina Reiter, Silvia Budday

**Affiliations:** ^1^ Institute of Continuum Mechanics and Biomechanics, Friedrich-Alexander-Universität Erlangen-Nürnberg, Erlangen, Germany; ^2^ Ecole Polytechnique, Palaiseau, France

**Keywords:** active learning, neural network, surrogate model, parameter identification, human brain tissue

## Abstract

Inverse mechanical parameter identification enables the characterization of ultrasoft materials, for which it is difficult to achieve homogeneous deformation states. However, this usually involves high computational costs that are mainly determined by the complexity of the forward model. While simulation methods like finite element models can capture nearly arbitrary geometries and implement involved constitutive equations, they are also computationally expensive. Machine learning models, such as neural networks, can help mitigate this problem when they are used as surrogate models replacing the complex high fidelity models. Thereby, they serve as a reduced order model after an initial training phase, where they learn the relation of in- and outputs of the high fidelity model. The generation of the required training data is computationally expensive due to the necessary simulation runs. Here, active learning techniques enable the selection of the “most rewarding” training points in terms of estimated gained accuracy for the trained model. In this work, we present a recurrent neural network that can well approximate the output of a viscoelastic finite element simulation while significantly speeding up the evaluation times. Additionally, we use Monte-Carlo dropout based active learning to identify highly informative training data. Finally, we showcase the potential of the developed pipeline by identifying viscoelastic material parameters for human brain tissue.

## Introduction

Computational mechanics models are a versatile tool to predict the response of nearly arbitrary geometries under mechanical loading. Exemplary applications from the field of brain biomechanics are the simulation of the human head under impact (Ji et al., 2022), cortical folding during brain development (Garcia et al., 2018; Zarzor et al., 2021), and brain deformation during surgery (Safdar et al., 2023). All these models depend on the availability of constitutive models and corresponding parameters that accurately characterize the material behavior. Therefore, the reliable identification of these parameters based on experimental data is an important preliminary task. This is usually done by deforming a specimen of a known geometry in a controlled manner while recording the force response. In the case of inhomogeneous deformation states during testing, the geometry as well as the boundary conditions of the experiment can be replicated through a simulation model. Subsequently, the material parameters of this forward model are iteratively updated until the simulated response is close enough to the recorded response. This process is called inverse parameter identification. The computational costs of this task are mainly determined by the complexity of the used forward model. The time needed to identify parameters for analytical models is usually comparably low, while for high fidelity models, e.g., finite element (FE) models, the computational costs for an inverse parameter identification can exceed those of the application models for which the parameters are later used. Nevertheless, some experimental setups and the thereby induced deformation states make it necessary to use computational models and an inverse parameter identification scheme, e.g., due to non slipping boundary conditions by gluing (Voyiadjis and Samadi-Dooki, 2018; Felfelian et al., 2019; Budday et al., 2020).

Different approaches have been developed to mitigate the problem of costly parameter identification. For gradient-based optimizations, the adjoint method can be used to obtain gradients directly from the simulation as opposed to using finite differences (Chavent, 2010; Balaban et al., 2016). Other approaches, namely, reduced order models (ROM), reduce the complexity of the models to also reduce computational costs. A subset of these methods are invasive in the sense that they seek to modify the approximation of the described system, e.g., proper orthogonal decomposition (Soldner et al., 2017), reduced basis methods, or proper generalized decomposition (Chinesta et al., 2017).

In recent years, machine learning techniques have become increasingly popular, as they were successfully applied to a wide variety of problems and have also found multiple applications in the field of solid mechanics (Kumar and Kochmann, 2022; Brodnik et al., 2023), including parameter identification. Kakaletsis et al. (2023) tried to replace the whole identification routine with a neural network that was trained on pairs of simulation output and mechanical parameters but did not obtain satisfactory results. They achieved better results when they used a Gaussian process regression as a surrogate model or ROM to replace the finite element simulation in a least-square regression. This use case highlights the ability of machine learning methods to serve as nonlinear regression models in terms of ROMs without deeper understanding of the underlying physical principles (Mohan and Gaitonde, 2018; Zhuang et al., 2023). However, these methods usually lose the basic model structure and with that also inherent properties like stability or convexity. Still, they are a valid and useful tool to obtain first estimates in inverse identification tasks. A subsequent optimization with the high fidelity model can mitigate these drawbacks and increase the accuracy of the final identified parameters. Schulte et al. (2023) used a neural network to obtain first estimates for a mechanical characterization task and subsequently continued the optimization with a high fidelity finite element model.

Depending on the modeled constitutive behavior, different approaches have been shown to perform well. For hyperelastic metamodels, feed forward neural networks (Hinrichsen et al., 2023a) and Gaussian process regression (Kakaletsis et al., 2023) were successfully used as surrogate models. Recurrent neural networks (RNN) have been shown to well capture time-dependent behavior, such as viscoelasticity (Chen, 2021) or plasticity (Gorji et al., 2020; Borkowski et al., 2022). This can be attributed to their ability of storing a persistent state, comparable to internal variables in constitutive models.

The aforementioned methods have in common that they rely on a preliminary training step where they “learn” the relation of inputs and outputs. Thus, the question of selecting appropriate samples for the training arises. The straightforward approach is the usage of random sampling or structured approaches like Poisson’s disk (Seo et al., 2023) or latin hyper cube sampling (Iordanis et al., 2022). In the present use case of surrogate models, the generation of training data is linked to computational costs in terms of simulation runs. Therefore, it is desirable to select the training points in such a way that the accuracy of the trained model is maximized. This is the aim of so called active learning approaches (Hasenjäger and Ritter, 2002). Several approaches have been developed for image classification tasks, e.g., BatchBALD (Kirsch et al., 2019), variational adversarial active learning (Sinha et al., 2019), and open-set recognition (Ren et al., 2021; Mandivarapu et al., 2022) gives a good overview over deep active learning approaches.

The so called acquisition function is used in active learning approaches to select the next best training points from a pool of unlabeled samples. A heuristic acquisition function that has been used successfully and can also be applied to nonlinear regression tasks is the estimated variance of the model (Kang et al., 2023). Different approaches have been developed to obtain this variance estimation. Some models like Gaussian process regression (Azizsoltani and Sadeghi, 2018) or Bayesian neural networks (Gal et al., 2017) directly report a confidence estimation together with their predictions. For “classical” neural networks that do not include these estimations in their predictions, different approaches have been developed. In the query by committee approach, multiple models are trained simultaneously and the variance in their predictions is used (Burbidge et al., 2007). Neural networks with a dropout layer enable another scheme to obtain these estimates, as their outputs are stochastic and can be queried multiple times via Monte-Carlo methods to also get an estimate of the variance or uncertainty of predictions (Kang et al., 2023). Additionally, the dropout layer can be disabled in the final training run on the full dataset when variance estimates are no longer needed.

In this work, we train a recurrent neural network on time-dependent viscoelastic simulation output using Monte Carlo dropout based active learning and show that it can reproduce simulation results with high accuracy. Subsequently, we integrate the trained network as a surrogate model in an inverse parameter identification to obtain an initial guess of the optimal set of parameters. Finally, we apply our pipeline to real experimental data from the mechanical testing of human brain tissue (Hinrichsen et al., 2023b). We finally assess to what extent this versatile tool can reduce computational costs in material characterization tasks. Due to the initial training costs, which are mainly caused by the necessary simulation runs, the presented approach becomes especially relevant when material parameters are determined for multiple sets of experimental data, e.g., in the region-specific characterization of brain tissue.

## Methods

### Mechanical model

**FIGURE 1:**
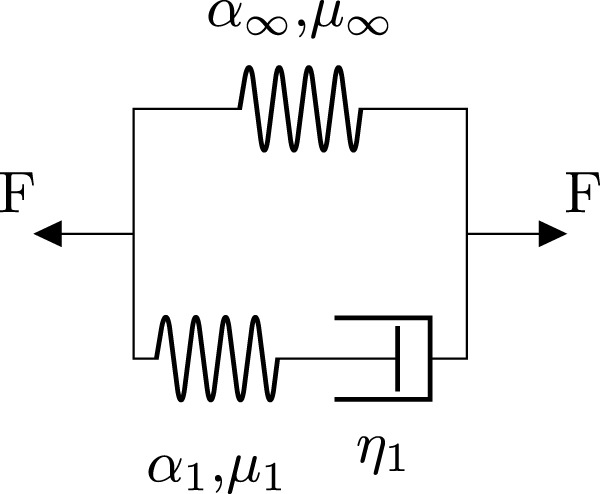
Rheological scheme of the implemented generalized Maxwell model with one Maxwell element. Each nonlinear spring represents a hyperelastic one-term Ogden model characterized by the shear modulus *μ* and the nonlinearity *α*. *η* denotes the viscosity of the dashpot element.

The high fidelity model that produces the ground truth solution for the neural network is a finite element simulation that implements a viscoelastic constitutive model. Figure 1 shows the rheological scheme for the used generalized Maxwell model consisting of a spring element, representing the equilibrium response, and one generalized Maxwell element with a nonlinear spring and dashpot.

We implement the finite vicoelastic model described in (Reese and Govindjee, 1998; Budday et al., 2017b) that is based on a multiplicative split of the deformation gradient **F** into an elastic and a viscous contribution. The deformation gradient can be obtained as the gradient ∇_**X**_ *φ*(**X**, *t*) of the deformation *φ*(**X**, *t*), which maps from the material configuration **X** to the deformed configuration **x** at time *t*. The multiplicative split is then written as

$$F=F^{e}\cdot F^{v},\;$$ (1)

where the superscripts *e* and *v* denote the elastic and viscous contributions, respectively. Furthermore, we can write the viscoelastic strain energy as sum of an equilibrium and non equilibrium parts, where the latter contains the contributions of the generalized Maxwell element(s)

$$\psi =\psi^{\text{e}q}+\psi^{\text{n}eq}$$ (2)

Each spring is described by a compressible one-term Ogden model (Ogden, 1972; Holzapfel, 2000; Hinrichsen et al., 2023b) defined by the strain energy function

$$\Psi =\Psi_{\text{iso}}+\Psi_{\text{vol}}\;with$$ (3)

$$\Psi_{\text{iso}}=\frac{2\mu }{\alpha^{2}}\left({\bar{\lambda }}_{1}^{\alpha }+{\bar{\lambda }}_{2}^{\alpha }+{\bar{\lambda }}_{3}^{\alpha }-3\right)\;and$$ (4)

$$\Psi_{\text{vol}}=\kappa \frac{1}{4}\left(J^{2}-1-2lnJ\right)$$ (5)

Where ${\bar{\lambda }}_{a}=J^{-1/3}\lambda_{a}$ are the isochoric principal stretches. *J* = det(**F**) denotes the volume ratio. The one-term Ogden model was shown to well capture the mechanical behavior of human brain tissue, especially the pronounced compression-tension asymmetry (Budday et al., 2017b; Hinrichsen et al., 2023b; Budday et al., 2017c). Hence, each spring is characterized by the shear modulus *μ*–we adapt the modified formulation in Budday et al. (2017a)–and the nonlinearity parameter *α*. The bulk modulus *κ* characterizes the compressibility in both springs but is not a free parameter as we calculate it from the shear modulus *μ* using the relation

$$\kappa =\mu \frac{2\left(1+\nu \right)}{3\left(1-2\nu \right)}$$ (6)

from the linear regime, where we use the Poisson’s ratio *ν* = 0.45. In our previous work (Hinrichsen et al., 2023b), we have identified hyperelastic parameters for Poisson’s ratio values *ν* = {0.45, 0.49} with no significant differences in the obtained fitting error. We assume purely deviatoric contributions to the viscosity and therefore set *η* = *η*_*d*_ and *η*_*v*_ = *∞* in the formulation by Reese and Govindjee (1998). The latter is achieved by setting $\frac{1}{9\eta_{v}}=0$ in Equation (45) of their work. We denote the parameters of the equilibrium spring and the Maxwell element with the subscripts *∞* and 1, respectively. Subsequently, the set of parameters that needs to be determined includes *μ*_*∞*_, *μ*_1_, *α*_*∞*_, *α*_1_, and *η*_1_. We implement the model in C++, using the finite element library deal.ii (Arndt et al., 2021).

### Experimental data

**FIGURE 2:**
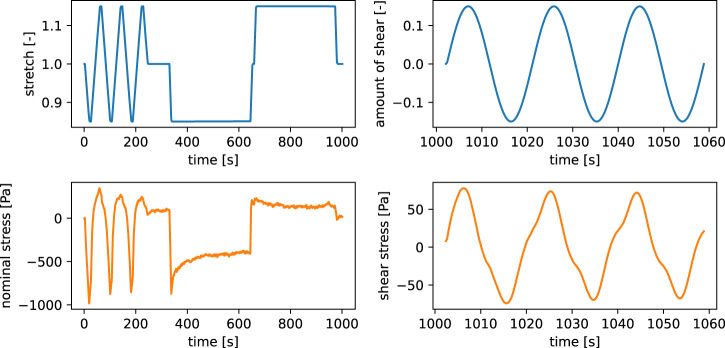
Experimental data of a human brain tissue specimen from the frontal cortex.

To test our developed hybrid inverse viscoelastic characterization approach, we use experimental data from a human brain tissue sample of the frontal cortex. Detailed information regarding the preparation of the human brains as well as the used testing protocol are described in (Hinrichsen et al., 2023b). The specimen was tested under cyclic loading and stress relaxation in compression-tension, and subsequently also under cyclic torsional shear. In this setup, the rheometer imposes a prescribed time-strain curve upon the tested specimen and records the response in terms of axial force *f*_*z*_ and torque *t*. To obtain a geometry-independent measure, we calculate the nominal stress *P* as well as the shear stress *τ* from the axial force *f*_*z*_ and the torque t as *P* = *f*_*z*_/*A* and *τ* = 2*t*/*πr* ^3^, where *A* denotes the undeformed cross sectional area and *r* the radius of the cylindrical samples. The data is subsequently filtered using a moving average as well as a Ramer-Douglas-Peucker Douglas and Peucker (1973) filter. Figure 2 shows the resulting time-series curves.

### Inverse parameter identification

Mechanical characterization of a material using a constitutive model and experimental data translates to an optimization problem, where the error between experimental data and model prediction needs to be minimized. Here, we benchmark our presented approach by characterizing the mechanical behavior of human brain tissue. In the following, we use *y* as a placeholder for the nominal stress P for compression/tension and the shear stress *τ* for torsional shear loading, respectively. We measure the error between the simulated values $y_{i}^{\text{s}im}$ obtained from the finite element or surrogate model and the experimental values $y_{i}^{\text{e}xp}$ as normalized L2 norm

$$\chi^{2}=\frac{\sum_{i=1}^{N}{\left(y_{i}^{\text{exp}}-y_{i}^{\text{sim}}\right)}^{2}}{\sum_{i=1}^{N}{\left(y_{i}^{\text{exp}}\right)}^{2}},$$ (7)

which is adapted from Gavrus et al. (1996). The optimization problem of finding the optimal material parameters that minimize *χ* ^2^ is then solved using the trust region reflective algorithm in the Python library scipy (Virtanen et al., 2020). We obtain the gradient that is needed for the optimization via finite differences.

The optimization is initially started with the recurrent neural network surrogate model. After an optimal set of parameters that minimizes the L2 error between experimental data and simulation output has been found, we use these parameters to restart the optimiziation with the high fidelity finite element model. Thus, we can use the fast evaluation times to get a good initial estimate and subsequently need less computationally expensive evaluations of the high fidelity model. A benefit of the trust region reflective algorithm is its ability to also handle constrained optimization problems. We utilize this to constrain the input of the finite element model to physically valid parameters, i.e., positive shear moduli *μ* and viscosities *η*. Additionally, we constrain the optimization on the surrogate model to parameter values inside the range of the training data. Thus, the model is prevented from extrapolating, which can be problematic for neural networks (Kim et al., 2021; Muckley et al., 2023).

### Recurrent neural network surrogate model

**FIGURE 3:**
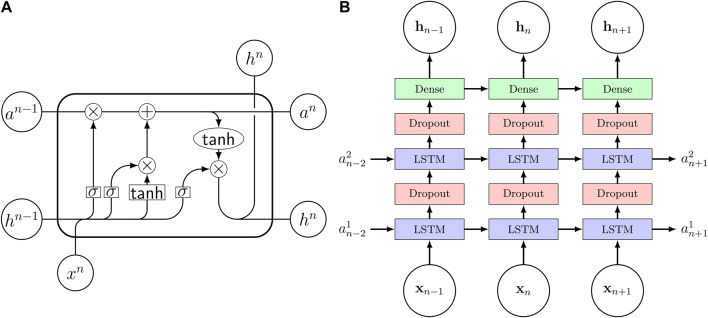
**(A)** Signal flow through an LSTM cell. *σ* denotes the sigmoid activation function. **(B)** Recurrent neural network architecture consisting of three layers with long short-term memory (LSTM) cells and two dropout layers in between. The input vector **x** contains the constitutive parameters as well as strain and time while the output **h** is the nominal stress **P** and shear stress *τ*. The internal cell state is stored in the state vector **a** and the subscript *n* denotes the timestep.

Viscoelastic materials show a history-dependent behavior, as their stress response is not only dependent on the currently applied strains but also on the loading history. In computational models this characteristic is captured by internal variables describing the internal state of the material. Chen (2021) pointed out the similarities between the structure of viscoleastic constitutive models and recurrent neural networks (RNN) making the RNNs a promising candidate to model viscoelastic behavior. Furthermore, they trained a RNN on 3D synthetic uniaxial loading data and showed good performance when the model was used to predict data not used during training. The RNN consisted of two layers, each containing 50 long short-term memory (Hochreiter and Schmidhuber, 1997) (LSTM) cells. Figure 3A shows the signal paths through an LSTM cell with a tanh function. Motivated by these results, we use a RNN as surrogate model to approximate the relation of in- and outputs of a high fidelity viscoelastic finite element simulation and thereby speed up the viscoelastic characterization from experimental data. Figure 3B visualizes the network architecture. We use two hidden layers, each consisting of 64 LSTM cells, followed by a dense output layer with two nodes. Additionally, we use two dropout layers in between the hidden LSTM layers to enable our Monte-Carlo dropout-based active learning strategy. The dropout rate of the two layers is set to 0.5 during the active learning phase. After all training points have been identified, the model is trained for one more time with the dropout rate set to 0, effectively disabling the dropout layer. To avoid numerical problems, we offset and scale the inputs and outputs so that the thereby normalized quantities are obtained as

$$v_{\text{scaled}}=\frac{v-\mu^{\text{train}}}{\sigma^{\text{train}}},$$ (8)

where *μ* ^train^ and *σ* ^train^ denote the mean and standard deviation of the training data, respectively. The inverse of this transformation is again applied to the outputs of the model. For the training of the model, we use the mean absolute error (MAE) as loss function during training and ADAM as optimizer with a learning rate of 0.001. The model is trained on the same strain-time series with a fixed time step but varying material parameters. Thus, input data containing different time steps than the training data should be interpolated to the timestep size of the training data. We implemented the training and evaluation of the model in Python, where we use the recurrent neural network and ADAM implementations provided by the tensorflow module (Abadi et al., 2015).

### Dropout-based active learning

Algorithm 1 Active learning.

 **Require:** n,m
   initialize training data **T** using sampling method (e.g., Poisson Disk, random)
   **while** size(**T**) ≤ n **do**
     train model
     sample evaluation points **E**
     estimate model variance at **E**
     sort **E** by estimated variance
     initialize candidates **C**
     **for** i = 1 … m **do**
      select first point **p** from **E** satisfying distance*(**p**, **T**) ≤ min (1/length(**T**),min_dist**(**T**))
    add **p** to candidates **C**
   **end for**
   run simulation label(**C**) and add output to **T**
   train new model on **T**
 **end while**
 train the model once more on **T** for the final number of epochs with dropout rate = 0
 * distance in normalized parameter space
  ** minimum distance between points of dataset

As opposed to the uninformed generation or selection (sampling) of training points, active learning seeks to identify the set of training points that is expected to be most informative in terms of the obtained model accuracy (Hasenjäger and Ritter, 2002). Here, we select training points for the current model iteratively and “online”, during the training itself. Thus, the training is started with a small initial set of points in the input parameter space on which the model is trained. Subsequently, the active learning algorithm determines the next best set of parameters, i.e., those that will increase the model’s accuracy the most. We adopt the heuristic that the next optimal training point is the one with the highest prediction uncertainty of the model (Tsymbalov et al., 2018). Furthermore, we estimate this uncertainty with repetitive calls to our RNN that expresses stochastic behavior due to the used dropout layers. This approach has already been used successfully for the active training of neural networks for image segmentation (Kang et al., 2023) and regression (Tsymbalov et al., 2018), where it was presented as Monte-Carlo dropout-based active learning. We note that we also investigated the alternative approach of training multiple individual models and querying the variance of their predictions. Preliminary investigations showed a similar performance as the dropout layer approach, while the computational costs for the training significantly increase with the used number of models.

Algorithm 1 describes the implemented active learning algorithm. We start with an initial set of training points and iteratively evaluate the model variance on a separate evaluation set. This set is sampled in each iteration, where we then select the points that satisfy a distant constraint relative to all points in the current training set. If no point is found that satisfies this constraint, the next one in the evaluation set, sorted by the variance, will be selected nonetheless. This is done until a preselected number of points is found and the next iteration starts. After a preselected number of iterations, the model is trained once more on the final set of training data with the dropout layer turned off (dropout rate set to 0). One would usually use a lower number of epochs in the intermediate training steps and subsequently train the final model for more epochs to achieve a good compromise between accuracy and computational effort.

## Results

### Dropout layer based active learning speeds up surrogate model training

**FIGURE 4:**
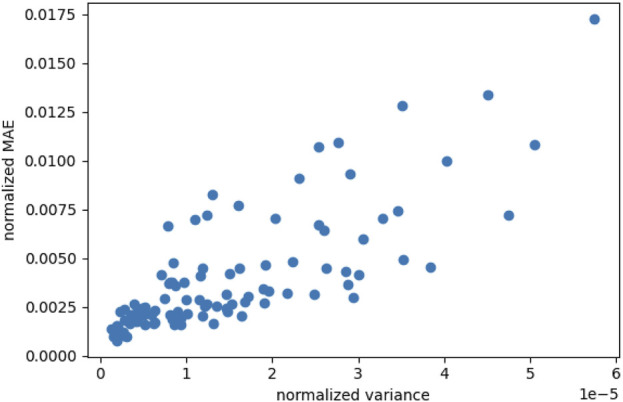
Correlation between the variance obtained by Monte Carlo dropout sampling and the mean absolute error (MAE) where both are normalized by their standard deviation over all samples. Results are shown for 100 points in the material parameter space and the same time series input (shear and stretch) that were created using Poisson Disk sampling.

Our employed active learning approach relies on the assumption that the variance of a neural network with dropout layers can indicate the most rewarding locations in the input parameter space in terms of gained prediction accuracy. To investigate the validity of this assumption, we evaluate the variance in the predictions of our model as well as the achieved accuracy in terms of mean absolute error (MAE) to the finite element simulation, i.e., the ground truth. To this end, we trained the network for 100 epochs on 25 points obtained via Possion Disk sampling, where the implementation of the latter is taken from the Python module scipy (Virtanen et al., 2020). Subsequently, the model was evaluated on a separate test set of 100 points. Figure 4 shows a clear trend between the variance of multiple calls to the model, using the same input parameters, and the MAE. The model was called 16 times and the MAE was calculated for the mean of these predictions.

**FIGURE 5:**
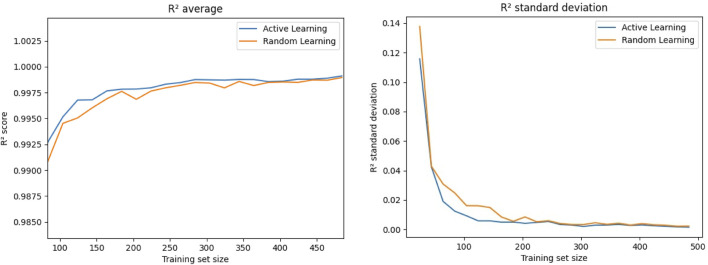
Comparison between the value as well as the standard deviation of the determination coefficient *R* ^2^ for adding new points to the training data set when they are selected randomly or via active learning, e.g., those with the highest estimated variance.

Next, we tested the performance of the active learning approach. Initially, we trained our recurrent neural network on 24 training points and subsequently retrained the model for 100 epochs after adding 4 additional points following Algorithm 1. We repeated this step 119 times, thus ending up with 500 training points after the final iteration. Additionally, we used a “random learning” approach with new training points being selected randomly. For the evaluation of these approaches, we copy the model every five iterations, e.g., after adding 20 training points, and train it for 1,000 epochs, as this would also be the procedure for the final selected training set. Figure 5 shows the average of the determination coefficient *R* ^2^ as well as its standard deviation over the training set size. We observe consistently better predictions from the model that was trained using active learning, indicated by the higher *R* ^2^. The standard deviations of *R* ^2^ are also lower for the active learning approach, indicating a better consistency in the prediction accuracy. Nevertheless, the shown curves indicate that the biggest differences are found for smaller training sets, followed by an asymptotic behavior with both approaches yielding similar values for later iterations and therefore higher training set sizes.

### Recurrent neural networks can approximate a viscoelastic finite element simulation

**FIGURE 6:**
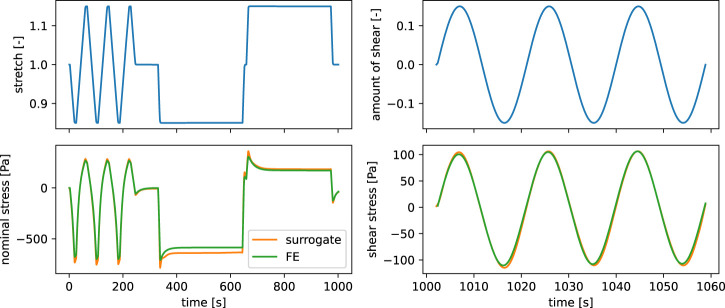
Comparison of metamodel and finite element simulation output in terms of nominal and shear stress for the same time series input (stretch and shear stress) as well as identical material parameters.

The main requirements for our surrogate model are a reduction of the computational costs together with a high accuracy in terms of differences in the output from the high fidelity model. Here, we analyze the performance of the final surrogate model that has been trained for 1,000 epochs on 500 training points, selected via the Monte-Carlo dropout-based active learning. A comparison of the output of both models in Figure 6 shows that the RNN model is able to well reproduce the qualitative behavior of the viscoelastic finite element simulation output. The models were both run on continuous strain input data of cyclic and stress relaxation loading in compression-tension as well as cyclic torsional shear loading, as introduced in Section Experimental data. Still, we observe some deviations in the maximum amplitudes for the cyclic and stress relaxation loading in compression, while especially the behavior under cyclic torsional shear is well reproduced. The computational costs in terms of cpu time for the shown predictions, measured with the command line utility time under Ubuntu 22.04, are 13 m 49.8 s and 8.4 s for the finite element simulation and the surrogate model, respectively.

Subsequently, we evaluated the model on a test data set containing 1,000 points and obtained an average *R* ^2^ value of 0.9985 as well as a standard deviation of 0.0024, indicating a robust prediction behavior with consistently good results.

### Surrogate modeling accelerates parameter identification

Our main motivation for the presented active learning surrogate modeling approach is the speed up of inverse parameter identification tasks. We benchmark our trained RNN surrogate model on the viscoelastic characterization of human brain tissue from experimental data described in Section Experimental data. As the response of a viscoelastic material depends on its loading history, we pass the whole strain-time-series (axial stretch and amount of shear) prescribed by the rheometer as input to the models. Subsequently, we obtain also the output as time-series data of nominal stress *P* and shear stress *τ*.

**FIGURE 7:**
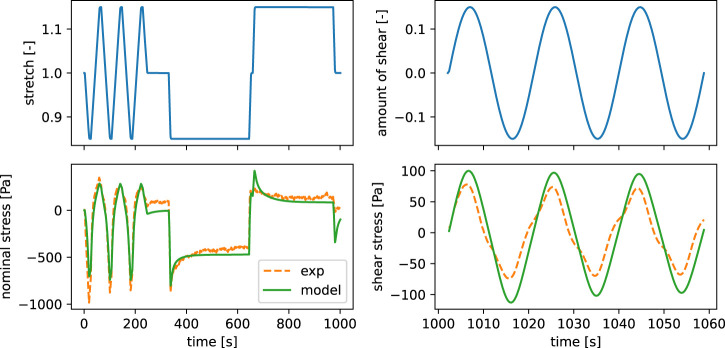
Parameter identification results for experimental data of human brain tissue from the frontal cortex. The finite element simulation output is shown for the final identified parameter set with *α*_*∞*_ = −16, *μ*_*∞*_ = 162 Pa, *η*_1_ = 13,949 Pa⋅s, *α*_1_ = −18, *μ*_1_ = 398 Pa.

Figure 7 shows that the inverse parameter identification is able to find a reasonably well fitting set of material parameters for experimental data of human brain tissue. The model shows a slight underestimation in compression during cyclic compression-tension in terms of the predicted nominal stress, while we observe an underestimation of the shear stresses during cyclic torsional shear loading. Still, the predicted stresses during stress relaxation in compression and tension show good agreement with the experimental data.

**FIGURE 8:**
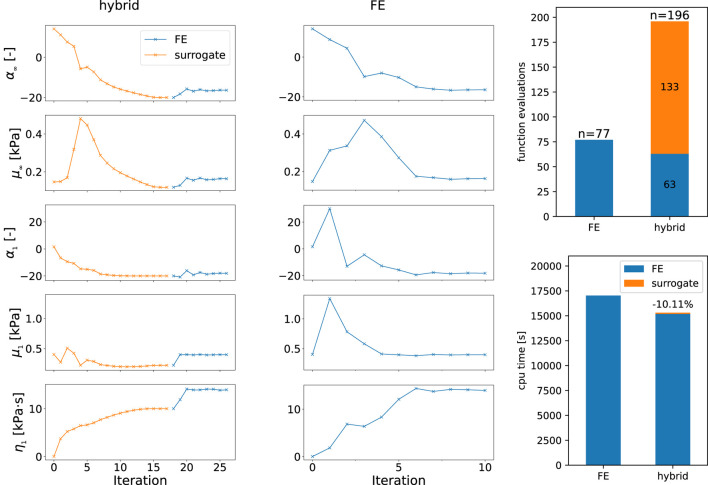
History of parameter values during the optimization using the hybrid approach with the surrogate and finite element model combined (hybrid) as well as only the finite element model. Function evaluations contain all model evaluations including finite difference approximations of the gradient as well as failed optimization steps. The cpu times are measured using the perf_counter function of the Python module time.

Figure 8 shows the optimization history in terms of parameter values found by the optimization that reduce the error of the previous iteration. Thus, we can compare the hybrid approach, where the optimization problem is initially solved for the surrogate and then the finite element model, as opposed to using only the finite element model from the start. The optimization is constrained by the boundaries in Table 1 that are defined for the two models separately. Here, the boundaries for the finite element simulation are also used in the second phase of the hybrid approach. We switch from the surrogate to the finite element model when the optimization of the surrogate model output has fully converged. We observe that the nonlinearities *α* as well as the viscosity *η* continuously approach the final values and show reasonable first estimates. The shear moduli *μ* show initial oscillations for *μ*_1_, while *μ*_*∞*_ rises in the first iterations until it reaches a noticeable peak and continuously decreases to the final value that underestimates the later found optimal value. We note that the values obtained from the optimization of the surrogate model for *α*_*∞*_, *α*_1_, and *η*_1_ assume the values of their prescribed upper boundaries in Table 1, while *μ*_*∞*_ is close to its lower boundary. Nevertheless, the number of function evaluations and the used CPU times in Figure 8, with ∼4h15 m and ∼4h44 m for the finite element and hybrid approach, respectively, show how the low computational costs of the surrogate model can help to accelerate parameter identification tasks. The break-even point, where the accumulated saved computational effort due to the surrogate-based approach is higher than those for the initial training is difficult to calculate, as it depends on the computational setup and the differences will certainly vary between different fitted datasets. But as an approximation based on the shown example with 14 saved simulation runs, one would have to fit 36 experimental curves to overcome the initial effort needed to train the 500 points used for training, neglecting the effort for the network training itself.

**TABLE 1:** Boundary constraints used during the optimization of the surrogate recurrent neural network model and the finite element simulation.

Parameter	RNN surrogate	FE simulation
*α*_*∞*_ [-]	[-20,20]	[-100,100]
*μ*_*∞*_[Pa]	[100,2000]	[0,*∞*)
*η*_1_ [Pa⋅s]	[0,10^4^]	[0,10^5^]
*α*_1_ [-]	[-20,20]	[-100,100]
*μ*_1_ [Pa]	[100,2000]	[0,10^4^]

## Discussion

In this work, we show how a recurrent neural network (RNN) surrogate model can speed up viscoelastic inverse material parameter identification based on finite element simulations. In a first step, we have quantified the accuracy of the RNN surrogate model in comparison to our ground truth finite element simulation. Additionally, we have demonstrated how Monte-Carlo dropout based active learning can accelerate the training process by selecting the training points with highest uncertainty from a sampled set of candidates. Finally, we have employed our trained model for the viscoelastic characterization of a human brain tissue sample based on experimental data from cyclic loading and stress relaxation in compression and tension as well as cyclic torsional shear. A comparison of our proposed hybrid approach, initially starting with the surrogate model and then switching to the finite element simulation, to the inverse identification using only the finite element model shows the capability to reduce the computational effort.

### The advantage of using recurrent neural networks as surrogate models

We have trained the recurrent neural network in Figure 3 on in- and output data of viscoelastic finite element simulations to obtain a surrogate - or reduced order - model. The final model shows good performance as it is able to reproduce the finite element simulation output with high accuracy (Figure 6). This is in good agreement with the results reported by Chen (2021) who trained a recurrent neural network containing long short term memory cells on output data from an analytical viscoelastic model, assuming infinitesimal strains and linear isotropic constitutive behavior. Similar to their work, we have used a many-to-many architecture with a fixed number of time steps during training. Nevertheless, this limitation can be easily circumvented by the interpolation of input data to the needed format. Additionally, our model is able to capture the highly nonlinear relation of in- and output data in the finite element simulations, which is caused by inhomogeneous deformation states due to non-slipping boundary conditions during testing (Hinrichsen et al., 2023b) as well as the nonlinear finite strain generalized Maxwell constitutive model. We note that this approach becomes computationally profitable when the accumulated time saved in each optimization becomes higher than the time needed for the initial surrogate model training.

### Monte-Carlo dropout-based active learning can speed up surrogate model training

The training of our recurrent neural network surrogate model is linked to significant computational costs due to the needed finite element simulation runs for the generation of ground truth labels. Hence, we have implemented a Monte-Carlo dropout-based active learning scheme (Algorithm 1) to iteratively update our training set with the next best points in the parameter space in terms of the estiamted gain in model accuracy. Here, we obtain this estimate in terms of the model uncertainty, which is again obtained by Monte-Carlo sampling of the stochastic neural network. We observe in Figure 5 that the active learning approach is able to improve the model performance in terms of achieved average *R* ^2^ and even more so for the standard deviation when we compare it to adding randomly sampled points from the parameter space. Still, we see an asymptotic behavior with both approaches, yielding similar results for later iterations. However, as the dominating costs of our surrogate model generation are those from data labeling in terms of finite element simulation runs, the additional effort for the active learning algorithm is from our perspective outweighed by the potential improvements for lower training set sizes. Similar results were achieved by others using dropout-based active learning (Tsymbalov et al., 2018).

### Use-case study: Viscoelastic parameter identification for human brain tissue

We have integrated the developed recurrent neural network surrogate model as a hybrid approach in our parameter identification pipeline, where we initially identify parameters for the surrogate model and use these as starting values for a subsequent optimization using the finite element simulation. The application of this approach to experimental data of human brain tissue in Figure 7 shows that we obtain good estimates for the optimal set of material parameters using the surrogate model. The comparison to an optimization on the same experimental data set and identical initial parameters (Figure 8) shows that the hybrid approach needs more iterations but is still noticeably faster.

### Limitations

An important limitation of purely data-driven methods like the here used recurrent neural network is their inability to capture the underlying physical principles, which can lead to unwanted behavior like unphysical solutions. Alternative hybrid approaches like constitutive artificial networks mitigate these limitations by the enforcement of physical constraints in their structure (Linka et al., 2021). Still, if the surrogate model is solely used to approximate an initial solution for parameter identification tasks, the appearance of such unphysical solutions will be less problematic: the validity of parameter values (e.g., positive shear moduli) can be enforced through optimization constraints.

As opposed to comparably simple analytical regression functions like polynomials, neural networks are practically a black box, as their sheer number of parameters prevents meaningful interpretations. This makes it difficult to estimate the model behavior for unknown input data and motivated us to constrain our model to the parameter ranges of the training data. New developments in the field of explainable machine learning (Adadi and Berrada, 2018; Muckley et al., 2023) have the potential to solve this problem in the future.

## Conclusion

In the present study, we have trained a recurrent neural network as surrogate model for viscoelastic finite element simulations. The trained model is able to well approximate the relation of in- and output data of the high fidelity finite element simulation for the same boundary conditions and strain-time-series but varying material parameters. Additionally, we have implemented a Monte-Carlo dropout active learning scheme that can guide the model training process by estimating the most rewarding training points to be added to the training set from the material parameter space. While this approach causes minimal computational overhead, we observed higher accuracy as well as decreased standard deviations of *R* ^2^ values in model predictions compared to random sampling for the same training set size. Although the improvements compared to plain random sampling become increasingly small for higher training set sizes, they are always present and thus motivate the general application of this method due to the mentioned acceleration potential. Finally, we have demonstrated the performance gains when using the surrogate model in a hybrid approach, where parameters are initially identified on the surrogate model yielding a good initial estimate and subsequently on the finite element simulation to achieve a higher accuracy. The results highlight the potential of applying the presented surrogate model architecture and training approach as a versatile tool to reduce computational costs in mechanical characterization tasks on the same experimental setup and protocol but varying samples.

## Data Availability

The original contributions presented in the study are included in the article/Supplementary Material, further inquiries can be directed to the corresponding author.
